# Intragenic CpG Islands and Their Impact on Gene Regulation

**DOI:** 10.3389/fcell.2022.832348

**Published:** 2022-02-11

**Authors:** James A. Cain, Bertille Montibus, Rebecca J. Oakey

**Affiliations:** Department of Medical and Molecular Genetics, King’s College London, Guy’s Hospital, London, United Kingdom

**Keywords:** polyadenylation, epigenetics, DNA methylation, orphan CpG-Islands, CpG island (CGI), alternative polyadenylation (APA), mRNA processing

## Abstract

The mammalian genome is depleted in CG dinucleotides, except at protected regions where they cluster as CpG islands (CGIs). CGIs are gene regulatory hubs and serve as transcription initiation sites and are as expected, associated with gene promoters. Advances in genomic annotations demonstrate that a quarter of CGIs are found within genes. Such intragenic regions are repressive environments, so it is surprising that CGIs reside here and even more surprising that some resist repression and are transcriptionally active within a gene. Hence, intragenic CGI positioning within genes is not arbitrary and is instead, selected for. As a wealth of recent studies demonstrate, intragenic CGIs are embedded within genes and consequently, influence ‘host’ gene mRNA isoform length and expand transcriptome diversity.

## Introduction

**FIGURE 1:**
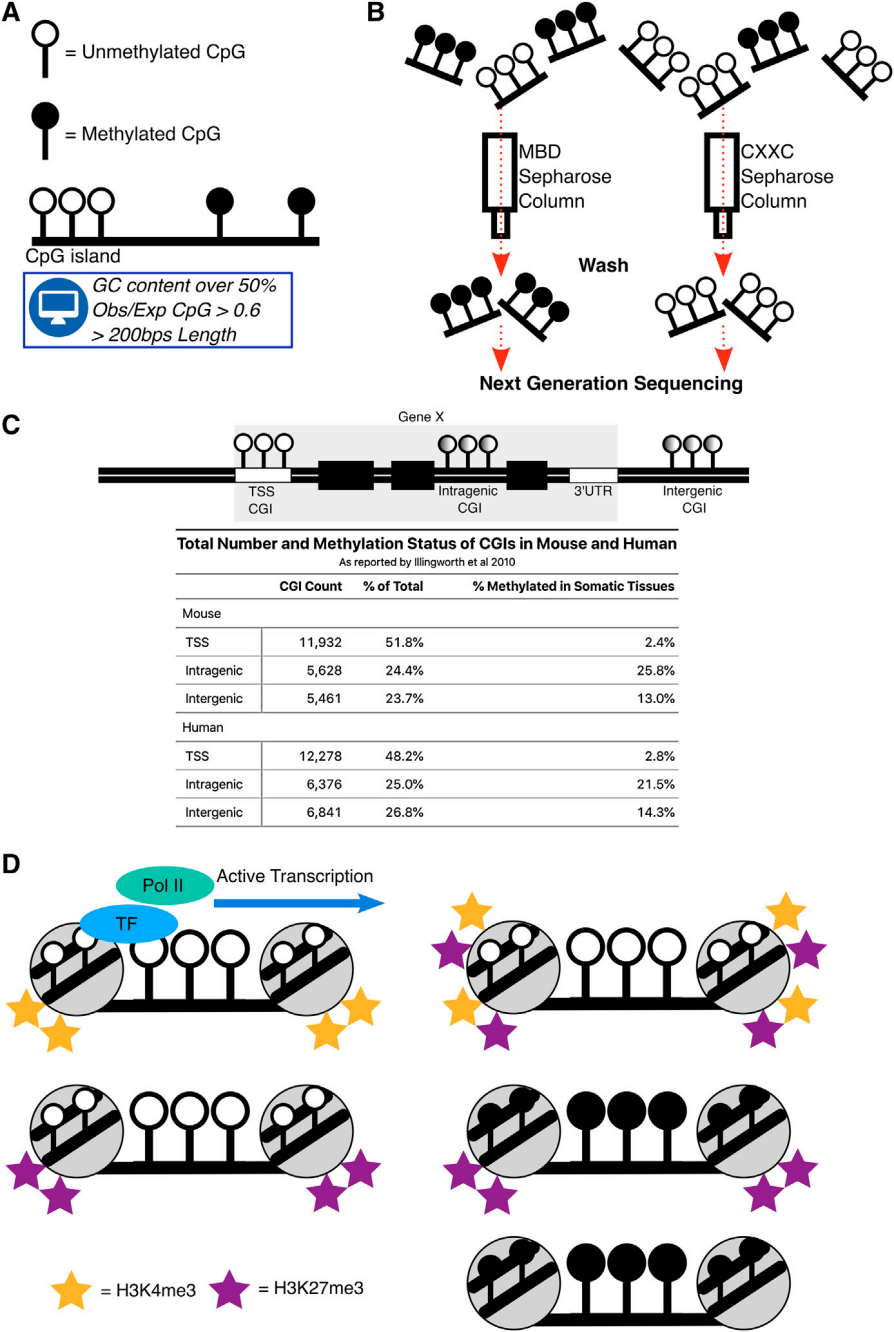
Definitions and states of CpG islands. **(A)** Depiction of the typical CpGs in the mammalian genome which are DNA methylated in isolation but are devoid of this mark in the CGI context. CGIs were originally defined bioinformatically. **(B)** Schematic demonstrating CGIs locations were biochemically determined. MBD and CXXC proteins fixed to a Sepharose column allowed purification of DNA methylated and unmethylated CGIs in mammals. **(C)** Representation of CGIs across mammalian genomes and a table, summarising the proportion and methylation status of CGIs as reported by Illingworth et al. (2010) in mouse and human across TSS, Intragenic and Intergenic regions. Total CGI numbers in mouse = 23,021, human = 25,495 **(D)** Summary of the main states of CGIs across the genome. Their active state is associated with binding of transcription factors (TF) and subsequent RNA Polymerase II (Pol II) binding. Repressed states of CGIs are through combinations of DNA methylation, H3K4me3 and H3K27me3.

Gene regulation is a prerequisite of life, the seemingly simple decision of whether to express a gene or not is present in nearly all organisms. Regulatory elements are sequence specific motifs in the mammalian genome that coordinate gene expression. One fundamental class of regulatory element are the CpG islands (CGIs). CGIs are regions of the genome that are enriched for cytosine and guanine dinucleotides (CpGs) and have been defined bioinformatically as having a GC content over 50%, an observed CpG ratio compared to the whole genome (Obs/Exp) of over 0.6 and a length of over 200 bps (Gardiner-Garden and Frommer, 1987) (Figure 1A). CpG’s in isolation are modified with DNA methylation, the addition of a methyl group onto cytosine, which is a heritable epigenetic mark. However, when CpGs congregate into islands they are generally protected from DNA methylation (Bird, 1978; Bird et al., 1979; Bird et al., 1985).

Instances where CGIs are DNA methylated have been correlated with transcriptional silencing when they are in close proximity to transcription start sites (TSS) of genes (Deaton and Bird, 2011). Using bioinformatic criteria, most CGIs are indeed found at TSSs, but this does not take into account their biochemical potential to exhibit or lack DNA methylation (Larsen et al., 1992; Takai and Jones, 2002; Saxonov et al., 2006). To overcome this, methods that specifically enrich for DNA fragments containing CGIs, with and without DNA methylation, were developed and combined with next generation sequencing to biochemically detect CGI coordinates (Illingworth et al., 2008; Blackledge et al., 2012) (Figure 1B). These studies found that only half of CGIs in mouse and human genomes are associated with TSS of genes and the rest are unannotated “orphans”, that are located either distal to genes (intergenic) or within genes themselves (intragenic) (Figure 1C). Not only are these “orphan” CGIs more likely to be DNA methylated (Figure 1C), but they are also more likely to demonstrate DNA methylation and transcriptional signatures in a tissue-specific manner (Illingworth et al., 2010; Deaton et al., 2011). Intragenic CGIs (iCGIs) are particularly striking as they are embedded within genes across mammalian species and are often not considered by most analyses, which use the standard bioinformatic definition of CGIs.

iCGIs can impact gene expression in a multitude of ways either by being transcriptionally active themselves or through interactions with biological processes in close vicinity. Biochemical methods discovered that a quarter of all CGIs are within genes, existing as iCGIs. Recent studies have now tied iCGIs to multiple functions (Maunakea et al., 2010; Jeziorska et al., 2017; Amante et al., 2020). Comprehensive reviews discuss CGIs more broadly and in the context of development and disease (Deaton and Bird, 2011; Greenberg and Bourc’his, 2019). This minireview aims to update the current knowledge of CGIs and capture their repertoire of functions outside of canonical TSSs.

## CGIs Are Promoters Independent of Genomic Position

Chromatin, the complex of DNA and histone proteins which forms chromosomes, can exist in an open or closed configuration indicative of active or inactive gene expression. The state of chromatin across the mammalian genome is studied through analysis of histone tail modifications, marking the histones that DNA is wrapped around. Over 100 histone modifications exist, some are well understood and some remain enigmatic, without a known biological function (Zhao and Garcia, 2015). Still, histone modifications are correlated to states of chromatin and are invaluable markers when studying gene regulation. CGIs overlap with >70% of canonical TSSs in the human genome and are typically associated with promoters (Saxonov et al., 2006), where they can exhibit multiple states, referred to here as ‘CGI states’. These states can be categorised depending on their histone marks.

One state is bivalency, where CGIs are transcriptionally repressed, devoid of DNA methylation and exhibit both active histone 3 lysine 4 trimethylation (H3K4me3) and repressive histone 3 lysine 27 trimethylation (H3K27me3) modifications. Bivalency has been proposed to poise CGI promoters for activation (Bernstein et al., 2006; Voigt et al., 2013), but has more recently been suggested to protect CGIs from DNA methylation whilst simultaneously keeping them transcriptionally inactive (Maupetit-Méhouas et al., 2016; Kumar and Jothi, 2020; Shah et al., 2021). The majority of promoter associated CGIs across the human genome exhibit bivalency (Court et al., 2019). This state likely arises due to the sequence composition of CGIs rather than their location, as CGIs experimentally introduced into the β-globin locus in mouse embryonic stem cells also displayed bivalency (Krebs et al., 2014; Wachter et al., 2014). The shift from bivalent CGIs to active CGIs is initiated through the binding of transcription factors, leading to the removal of H3K27me3, whilst maintaining the H3K4me3 mark. Removal of H3K4me3 and maintenance of H3K27me3 at the CGI is repressive, otherwise known as polycomb-only mediated repression, and is observed at a minority of promoter CGIs in somatic tissues (Mikkelsen et al., 2007; Farcas et al., 2012; Court et al., 2019; Blackledge et al., 2020).

A more stable form of repression at CGIs is through DNA methylation. In somatic tissues, DNA methylation represses promoter CGIs at the inactivated X chromosome (Augui et al., 2011; Galupa and Heard, 2018), germline genes (Velasco et al., 2010; Dahlet et al., 2021; Mochizuki et al., 2021), imprinted genes (Barlow and Bartolomei, 2014), and some lineage-committed genes (Dahlet et al., 2020). Whilst H3K27me3 and DNA methylation are both repressive, they are typically mutually exclusive at CGIs (Brinkman et al., 2012; Statham et al., 2012). Chromatin immunoprecipitation experiments indicate that H3K27me3 and DNA methylation can co-exist at some imprinted genes (Maupetit-Méhouas et al., 2016). CGIs can therefore exhibit multiple states of chromatin which are indicative of their transcriptional potential (Blackledge and Klose, 2011) (Figure 1D).

iCGIs are more likely to be DNA methylated (Figure 1C) and those lacking DNA methylation can exhibit bivalent chromatin signatures and when transcriptionally active, show transcription factor binding and the promoter mark, H3K4me3 (Lee et al., 2017; Amante et al., 2020; Choi et al., 2020). iCGIs can therefore exist in the same ‘states’ as promoter CGIs, albeit in different proportions. The iCGI states themselves are regulated in a tissue-specific manner, and with crosstalk from the gene that ‘hosts’ them. This can lead to both, consequences on the iCGI itself and their corresponding host gene.

## Consequences of Being an Intragenic CGI Within a Gene

**FIGURE 2:**
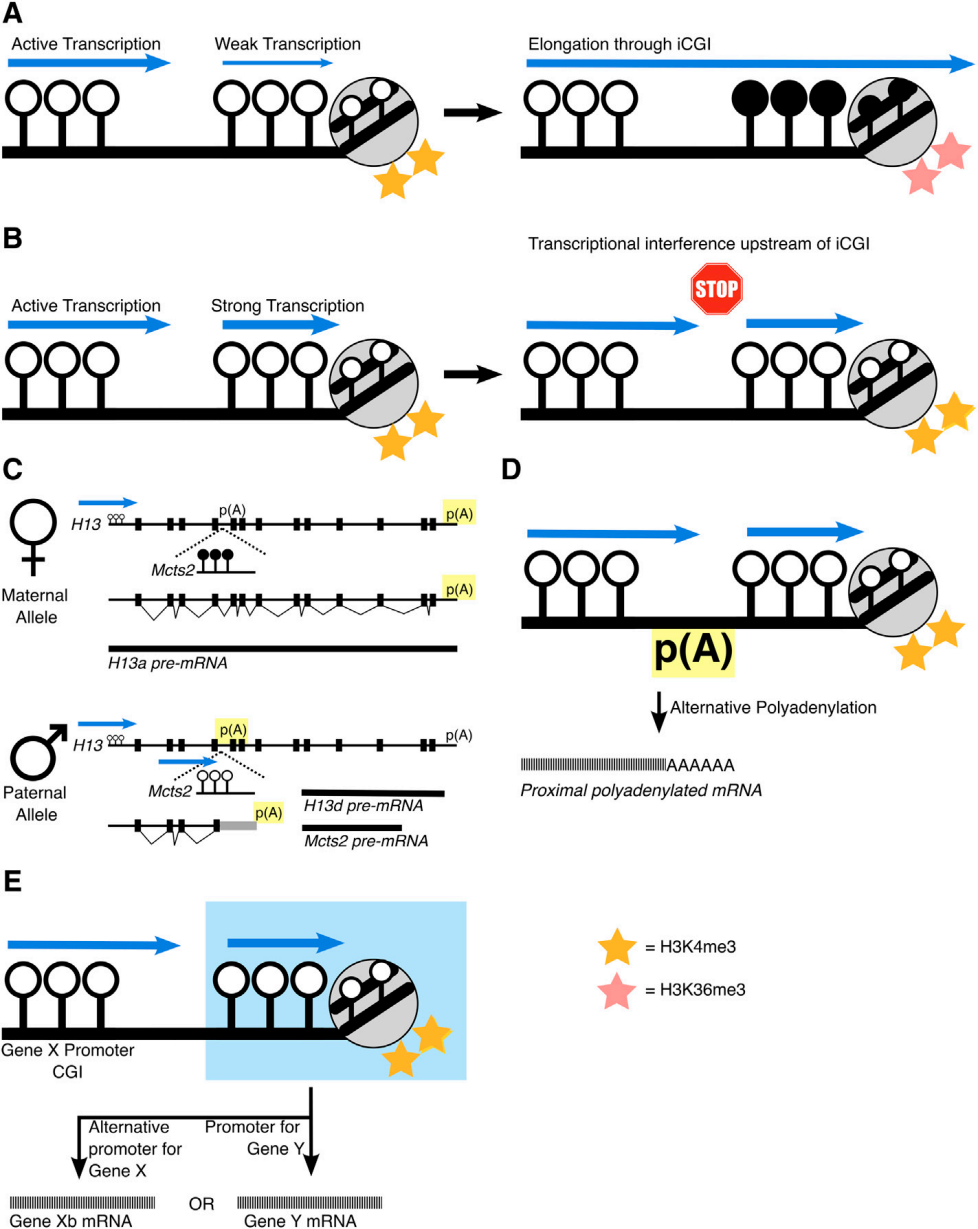
Schematics of how iCGIs impact gene regulation mechanisms. **(A)** Transcription through a ‘weak’ iCGI can silence it, depositing H3K36me3 and DNA methylation at the iCGI. **(B)** However, if the iCGI exhibits strong transcriptional activity, it can lead to transcriptional interference. This can result in events akin to those at the **(C)** H13/Mcts2 locus, that exhibits allele-specific PAS usage. Usage of the PAS is highlighted in yellow. **(D)** Similar mechanisms have been found other iCGIs. Alternatively, and in some cases, simultaneously, **(E)** the iCGI can act as a promoter itself, highlighted in blue, for either the host gene itself (gene X) or for a different ‘nested’ gene (gene Y).

The location of iCGIs within a gene is a turbulent place for a promoter region because active transcription results in the silencing of DNA which has been transcribed through. At first, this sounds paradoxical, but it has been identified at various loci that transcription through a gene promoter can silence it. This phenomenon was first demonstrated at the α-globin locus in a case of α-thalassemia where the *LUC7L* gene is juxtaposed upstream of *HBA2*. Here, *LUC7L* transcription extends through the *HBA2* promoter CGI which is subsequently DNA methylated and silenced (Tufarelli et al., 2003). This can be observed naturally at regions of genes that contain clusters of overlapping genes, such as at the imprinted loci, *Gnas* and *Igfr2* and likely, at *Kncq1* too. At the *Gnas* locus, incoming transcription from upstream *Nesp* removes H3K4me3 at the *Gnas* CGI and establishes DNA methylation and silencing (Chotalia et al., 2009; Williamson et al., 2011). Transcription of the *Airn* long non-coding RNA (lncRNA) through the *Igfr2* promoter silences *Igfr2* (Latos et al., 2012; Santoro et al., 2013). Similarly, the *Kcnq1* locus contains an overlapping transcript, *Kcnq1ot1*, that overlaps with the *Kcnq1* CGI promoter. Silencing of *Kcnq1* is correlated to transcription of the overlapping *Kcnq1ot1*, suggesting transcription itself is causing gene repression (Golding et al., 2011). Genome-wide analysis now highlights that this repression is through interactions between the transcribing RNA Polymerase II and the deposition of the elongation associated histone mark, H3K36me3. This in turn recruits DNMT3B, to deposit *de novo* intragenic DNA methylation (Baubec et al., 2015; Neri et al., 2017; Dahlet et al., 2020) (Figure 2A).

This may indicate that tissue-specific patterns of DNA methylation at iCGIs are a by-product of transcription through the gene itself, where iCGI function as a promoter is silenced when the host gene is transcriptionally active. Whilst iCGIs hosted within an active gene are generally silenced, subsets of iCGIs that show more RNA Polymerase II binding are protected from this silencing and maintain their H3K4me3 promoter status (Jeziorska et al., 2017). This indicates that iCGIs can resist the silencing power of transcription, but only if they are ‘strong’ enough to do so (Figures 2A,B).

What are the factors that dictate CGI strength? There is speculation that long CGIs may exhibit more sites for RNA Polymerase II binding (Elango and Yi, 2011) and a higher CpG density is correlated to enhanced transcription factor binding (Hartl et al., 2019). Given that iCGIs are generally shorter than promoter CGIs and less CpG dense, this may explain why a subset of iCGIs are silenced. But, despite this, subsets of iCGIs escape transcriptional silencing and this can have a series of effects on the host gene itself.

## Consequences on the Gene for Hosting an Active Intragenic CGI

Polyadenylation and splicing are co-transcriptional processes that can generate a diversity of mature mRNA isoforms from a single gene. Briefly, regulation of splicing and polyadenylation can control which exons of the pre-mRNA are utilised and when the pre-mRNA should be terminated. Alternative regulation of either of these processes impact the function of the mature mRNA (Proudfoot, 2011; Lee and Rio, 2015) and both recruit large protein machineries that regulate these processes co-transcriptionally (Lee and Rio, 2015; Tian and Manley, 2016; Gruber and Zavolan, 2019). As such, it seems plausible that iCGI activity can impact splicing and polyadenylation when they are co-occurring in close proximity.

Coincidentally, there are a wealth of studies linking active iCGIs to alternative polyadenylation (APA) events, specifically intronic APA (iAPA), which can alter the protein coding sequence of mRNA transcripts as it is terminated prematurely. This was first demonstrated at the imprinted *Mcts2/H13* locus (Wood et al., 2008). Here, *H13* isoforms are alternatively polyadenylated depending on the parental origin of DNA methylation at the iCGI within *H13*’s fifth intron. This iCGI is a promoter for a nested gene, *Mcts2* and when active (paternal allele), polyadenylation of *H13* occurs within intronic regions. However, when *Mcts2* and its iCGI promoter are silenced (maternal allele), polyadenylation occurs at the 3′UTR of *H13* (Figure 2C). This mechanism of APA is the same at the imprinted *Nap1l5/Herc3* locus. Here, an iCGI is a promoter for *Nap1l5* and its parental origin is correlated with the polyadenylation site choice of the host gene, *Herc3* (Cowley et al., 2012).

Outside of the imprinted context, two recent studies which perturbed DNA methylation showed similar results at iCGIs. Knockout of DNA methyltransferases (*DNMT1* & *DNMT3B*) in cancer cells increased initiating RNA Polymerase II at the iCGI which was correlated with the usage of proximal polyadenylation sites of two host genes (Nanavaty et al., 2020). Similar polyadenylation site usage was also found when DNA methylation was perturbed at the iCGI within the *NFATc1* locus, resulting in alternative *NFATc1* isoforms. These locus specific effects have been detected genome-wide by a recent bioinformatic screen, emphasising that iCGI activity leads to premature transcription termination upstream of the iCGI, most likely through APA (Amante et al., 2020) (Figures 2B,D).

These findings demonstrate that a transcriptionally active iCGI can influence alternative polyadenylation and highlight the ways in which iCGIs can shape the transcriptome. Mechanistically, this is likely due to RNA polymerase II prematurely stopping because of meeting another initiating polymerase at the iCGI, otherwise known as transcriptional interference (TI) (Shearwin et al., 2005) (Figure 2B). Here, the polyadenylation machinery selects the nearest site to avoid the production of an unstable mRNA transcript. It is still undetermined whether iCGI activity influences APA serendipitously through TI, or if this is a direct mechanism to regulate pre-mRNA termination.

An active iCGI can also influence isoform choice more directly, by acting as an alternative promoter for the host gene (Figure 2D). The *SHANK3* gene for example, contains an iCGI which is differentially methylated between hippocampus and cortex astrocytes. In hippocampus astrocytes, the iCGI is active and devoid of DNA methylation where it serves as an alternative promoter for *SHANK3*, transcribing a shorter mRNA transcript. Whereas in cortex astrocytes, when the iCGI is silenced through DNA methylation and instead, the canonical full length *SHANK3* isoform is transcribed (Maunakea et al., 2010).

## CGI Function as Enhancer Regions

Recent work suggests that CGIs may have another regulatory role as enhancers. Enhancers are *cis*-regulatory 50-150bp DNA sequences that are characterised by enriched transcription factor binding sites, H3K4me1 and H3K27ac histone modifications and when active, regions of bidirectional transcription produce enhancer RNAs (eRNAs) (Kim et al., 2010; Santa et al., 2010; Li et al., 2016). eRNAs confer *cis*-regulatory effects by recruiting transcriptional machinery to target genes to induce gene activation (Arner et al., 2015), otherwise referred to as enhancer looping. Intragenic enhancers can interfere with host gene expression through transcriptional interference (Onodera et al., 2012; Cinghu et al., 2017), similar to active iCGIs. Bioinformatic analyses show that iCGIs themselves exhibit enhancer histone modifications, are actively transcribed to eRNAs, are conserved across mammalian species (Bell and Vertino, 2017) and show greater transcription factor binding (Steinhaus et al., 2020). Such signatures have been identified at an iCGI within *Kdm6b,* which exhibits H3K4me1 and loops to the promoter CGI to enhance *Kdm6b* expression (Montibus et al., 2021). Given that transcription of enhancer regions is required to deposit enhancer histone marks, it is unclear how CGIs are initially defined as enhancer regions (Kaikkonen et al., 2013).

Enhancer signatures are also found at the other type of ‘orphan’ CGI, intergenic CGIs. A recent study has challenged the idea that these CGIs directly serve as enhancers, and instead, boost proximal enhancer function (Pachano et al., 2021). Here, intergenic CGIs augment enhancers’ ability to amplify only target genes that contain a CGI promoter themselves. As enhancers loop to promoter CGIs, the unmethylated intergenic CGIs that are within 3 kb of the proximal enhancer bring along machinery for efficient promoter CGI transcription. These intergenic CGIs also serve to protect transcription factor binding sites (TFBS) within the proximal enhancer from repressive DNA methylation (Pachano et al., 2021). The relationship between intergenic CGIs and proximal enhancers may be reciprocal, as the TFBS within the enhancer can assist recruitment of machinery to the intergenic CGI itself.

## Conclusions, the Relevance of Intragenic CGIS in Biology

CGIs are regions where transcription can initiate. Whilst most CGIs are localised to annotated TSSs, many can be found intragenically. In some cases, the iCGI is silenced; in others, active iCGIs impact pre-mRNA processing and promote or contain enhancer function.

iCGIs are more prone to DNA methylation during embryonic development and adult development compared to their TSS CGI counterparts (Illingworth et al., 2010; Auclair et al., 2014), implying that regulation of iCGIs is crucial for tissue specific programming. For example, iCGIs are specifically expressed in brain tissues and their host genes function in brain-specific biological processes (Amante et al., 2020). In this case, iCGIs may function as TSSs for novel transcripts or result in APA of the host gene and therefore, expand the transcriptome during developmental processes such as neurogenesis. iCGIs are conserved across mammalian species (Illingworth et al., 2010), suggesting they are maintained and necessary for proper gene regulation. It is still unclear how the multitude of functionalities of iCGIs are specified, i.e., how does an iCGI know to serve as an alternative promoter or, to disrupt host gene polyadenylation.

Similarly, DNA methylation of iCGIs prevents spurious intragenic transcription (Neri et al., 2017; Dahlet et al., 2020). Blocking spurious intragenic transcriptional activity is a method to ensure productive elongation by RNA polymerase II. DNA hypomethylation is widespread in cancer cells and extends to intragenic regions (Ehrlich, 2002; Hon et al., 2012; Kulis et al., 2012), hinting that intragenic transcription may be widespread in cancer (Kulis et al., 2013). The *RB1* gene, for example, contains an imprinted iCGI where its DNA methylation is inversely correlated to expression of the full length *RB1* transcript (Kanber et al., 2009; Kulis et al., 2012). The region which contains the iCGI is commonly deleted in cases of chronic lymphocytic leukaemia, implying that iCGIs may be disrupted in cancer cells. Despite this, intragenic DNA hypomethylation in cancer is mainly outside of promoter CGIs and they are paradoxically, hypermethylated instead (Kulis et al., 2013; Court et al., 2019). It is currently unknown if the cancer signature of hypomethylation extends to iCGIs, or if they are hypermethylated like promoter CGIs and if this is functionally relevant.

Given their distinct regulation and that many are protected from DNA methylation it is reasonable to suggest that iCGIs are required in mammalian biology. A clear challenge that has limited our understanding of iCGIs is their overlap with genomic annotations. Conventional short-read sequencing technologies present a challenge when trying to distinguish whether signals or reads stem from the host gene or the iCGI. The arrival of long-read sequencing technologies and the eventual decline in cost of such methods will allow these reads to be distinguished and aid understanding of iCGIs (Logsdon et al., 2020). This will further be enhanced by studying the methylation of iCGIs in more contexts, which will be possible when methods such as whole-genome bisulphite sequencing (WGBS) are more cost effective. Technology in its current state can also aid understanding of iCGIs, with greater reporting of genomic locations of CGIs in genome-wide analyses of DNA methylation, which are currently skewed to canonical TSSs.
